# Dmax-derived lactate-threshold intensity profiles and associated factors in adolescent swimmers: a multicenter cross-sectional study

**DOI:** 10.3389/fphys.2026.1935422

**Published:** 2026-08-31

**Authors:** Jiayi Lyu, Guangnan Liu, Jiawen Li, Lei Lei, Ze Zhang, Jie Gao

**Affiliations:** 1 School of Education, Beijing Sport University, Beijing, China; 2 School of Clinical Medicine, Chinese Academy of Medical Sciences & Peking Union Medical College, Beijing, China; 3 Yale College, Yale University, New Haven, CT, United States; 4 Beijing No.24 Middle School, Beijing, China; 5 Faculty of Arts and Education, The University of Auckland, Auckland, New Zealand

**Keywords:** adolescent athletes, individualized training, lactate threshold, latent profile analysis, swimming

## Abstract

**Objective:**

To identify data-driven Dmax-derived threshold-intensity profiles in adolescent swimmers by jointly considering pool-based and land-based incremental-test indicators and to examine hematological, performance-related, maturation-related, and psychoperceptual characteristics associated with profile membership.

**Methods:**

This multicenter cross-sectional study included 247 adolescent swimmers from three training centers. Participants completed a land-based cycling incremental test and a pool-based 5 × 200 m front-crawl incremental test. Pool-based Dmax-derived threshold intensity was expressed as swimming velocity at the Dmax point relative to event-specific personal-best velocity, whereas land-based Dmax-derived threshold power was expressed as cycling power output at the Dmax point. The two indicators were entered into latent profile analysis. Between-profile comparisons incorporated effect sizes, 95% confidence intervals, and Benjamini–Hochberg adjustment for multiple testing. Candidate variables were selected according to prior evidence and biological plausibility, screened for strong pairwise correlations, and entered into a multinomial logistic regression model with study center included as a categorical fixed effect.

**Results:**

Three ordered threshold-intensity profiles were identified: lower (n = 117, 47.4%), intermediate (n = 72, 29.2%), and higher (n = 58, 23.5%). The selected model showed good classification accuracy (entropy = 0.929). Relative to the lower profile, membership in the intermediate profile was associated with a higher red blood cell count (OR = 7.765, 95% CI: 3.198–18.849), lower perceived exertion (OR = 0.500, 95% CI: 0.372–0.672), and greater last-repetition completion relative to personal-best velocity (OR = 1.725, 95% CI: 1.317–2.259). Membership in the higher profile was associated with a higher red blood cell count (OR = 40.412, 95% CI: 10.844–150.605), lower perceived exertion (OR = 0.300, 95% CI: 0.197–0.457), greater last-repetition completion (OR = 2.903, 95% CI: 2.007–4.200), lower mental fatigue (OR = 0.954, 95% CI: 0.916–0.993), and greater sport mental toughness (OR = 1.134, 95% CI: 1.041–1.235).

**Conclusions:**

Adolescent swimmers exhibited three data-driven, ordered patterns in the joint distribution of pool-based Dmax intensity and land-based Dmax power. Profile membership was associated with concurrent hematological, performance-related, and psychoperceptual characteristics. These profiles provide an exploratory person-centered framework for describing cross-modal threshold-intensity heterogeneity rather than biologically discrete phenotypes or prescriptive training categories. Their reliability, longitudinal stability, and responsiveness to training require further evaluation.

## 1 Introduction

Accurate identification and control of training intensity are central to individualized training prescription, monitoring of training adaptation, and prevention of excessive load in competitive swimming (Skorski et al., 2012). Unlike land-based cyclic sports such as running or cycling, swimming performance is shaped not only by cardiorespiratory and metabolic capacity, but also by in-water technical factors, including stroke rate, stroke length, propulsive efficiency, and coordination (Ruiz-Navarro et al., 2025). This issue is particularly relevant in adolescent swimmers, whose age, sex, maturation status, training age, competitive level, and technical development may amplify individual differences in cardiorespiratory responses, lactate kinetics, technical economy, and perceived training load (Keller et al., 2023). These characteristics highlight the need for an individualized assessment framework that preserves the sport-specific relevance of threshold intensity while distinguishing the technical and physiological components of threshold performance.

Within this framework, pool-based lactate-threshold testing provides an ecologically relevant assessment of exercise intensity in the sport-specific environment. However, the resulting threshold velocity reflects the combined influence of metabolic capacity, hydrodynamic efficiency, stroke mechanics, and swimming-specific neuromuscular coordination, making it difficult to distinguish physiological capacity from technical proficiency on the basis of pool testing alone. A complementary land-based assessment may therefore provide additional information that cannot be obtained from swimming testing alone (Carvalho et al., 2020). Cycle-ergometer testing permits the external workload to be directly quantified and progressively increased under standardized conditions. Although cycling power is also influenced by body size, lower-limb muscle recruitment, and task familiarity, it is relatively less dependent on swimming-specific technique and can provide an additional perspective on threshold-related exercise capacity outside the technically complex aquatic environment (de Haan et al., 2024). Cycling-derived threshold power was therefore included not as a surrogate for swimming performance, but as a complementary indicator. Joint consideration of pool-based and land-based threshold intensities may identify concordant or discordant patterns across exercise modalities and provide a broader characterization of individual athletes than either assessment alone (Zamparo et al., 2020).

To obtain individualized threshold-intensity estimates using a methodologically consistent procedure across these complementary testing contexts, an approach applicable to both pool-based and land-based incremental tests was required. Blood lactate responses during incremental exercise reflect the changing balance between lactate appearance and removal as exercise intensity increases. Rather than determining threshold intensity from a predetermined blood lactate concentration, the Dmax method uses the shape of the individual’s complete lactate-intensity curve. After the curve is fitted, Dmax is identified as the point with the greatest perpendicular distance from the straight line connecting the first and final data points, and the corresponding swimming velocity or cycling power is taken as the individual threshold intensity (Cheng et al., 1992). This curve-based approach accounts for interindividual variation in baseline lactate concentration and the pattern of lactate accumulation, which may be particularly relevant in adolescent athletes with heterogeneous training backgrounds and maturation status (Nikitakis and Toubekis, 2021). Alternative contemporary threshold concepts include Critical Speed (CS)/Critical Power (CP), which define the boundary between heavy and severe exercise domains (Faude et al., 2009), and Maximal Lactate Steady State (MLSS), the highest exercise intensity at which blood lactate concentration remains stable over time. Compared with Dmax, CS/CP and MLSS typically require multiple constant-load trials on separate days (Poole et al., 2016), which can be logistically challenging in a multicenter adolescent sample. The Dmax method, in contrast, yields an individualized threshold estimate from a single incremental test per modality, making it a feasible choice for the present study design. In the present study, Dmax was selected because the same computational principle could be applied to both pool-based and land-based incremental tests while retaining modality-specific intensity outputs, swimming velocity and cycling power (Arsoniadis et al., 2024). In addition, an individualized threshold-intensity estimate could be obtained from a single incremental test in each modality, providing a feasible and methodologically consistent approach for the present multicenter sample. Accordingly, Dmax-derived intensity was used as an operational marker for characterizing cross-modal threshold-intensity profiles rather than as a direct measurement of a single physiological event.

When pool-based and land-based Dmax-derived intensities are considered jointly, traditional analytical approaches focusing on average between-group differences or isolated linear associations may not adequately capture distinct cross-modal patterns within athletes. Latent profile analysis is a person-centred, model-based approach that can identify unobserved subgroups from multiple continuous indicators and assign individuals to profiles probabilistically (Collins and Lanza, 2010; Nylund-Gibson and Choi, 2018). Compared with grouping athletes by fixed thresholds or arbitrary quantiles, latent profile analysis may be more appropriate for exploring heterogeneity in threshold intensity characteristics across different testing contexts. However, most swimming studies on lactate threshold assessment have focused on threshold determination methods, the validity of fixed lactate criteria, or the relationship between threshold velocity and technical variables (Espada et al., 2021). Fewer studies have used a person-centred approach to integrate pool-based sport-specific threshold intensity with land-based cardiorespiratory and metabolic testing indicators in adolescent swimmers.

Adolescent swimmers may exhibit distinct lactate threshold intensity patterns across pool-based sport-specific testing and land-based cycling testing because of individual differences in technical development, physiological adaptation, and perceptual regulation. We proposed two hypotheses:

Hypothesis 1 (H1) that adolescent swimmers would not present a single homogeneous lactate threshold intensity profile, but rather several latent profiles characterized by different intensity levels;

Hypothesis 2 (H2) that membership in a higher lactate threshold intensity profile would be associated with more favorable oxygen transport capacity, greater relative intensity tolerance, and more favorable perceptual responses.

Accordingly, this multicenter cross-sectional study aimed to identify lactate threshold intensity profiles in adolescent swimmers using pool-based Dmax-derived lactate threshold intensity and land-based Dmax-derived lactate threshold power through latent profile analysis, and to examine the associations of these profiles with hematological indicators, performance-related markers, and psychoperceptual markers. Given the cross-sectional observational design, the findings were interpreted as associations between profile membership and multidimensional monitoring markers rather than as evidence of causality or direct training prescription guidance.

## 2 Materials and methods

### 2.1 Design and sample

This was a multicenter, cross-sectional observational study. In the study, adolescent swimmers from three training sites in Beijing, China were enrolled by convenience sampling in May 2025 over a one-month period.

Inclusion criteria included: (i) age 10–19 years per the World Health Organization definition of adolescence; (ii) current registration and regular team training at one of the three centers; (iii) completion of the pool-based incremental swim test with blood lactate sampling; and (iv) athlete assent and parental/guardian consent as applicable. Exclusion criteria comprised: (i) discontinuation of team training during the study month; (ii) acute illness or musculoskeletal injury within the previous two weeks, or any medical condition contraindicating incremental testing; (iii) incomplete test procedures or missing key data (e.g., blood lactate, hemoglobin/red blood cell counts, or rating of perceived exertion); and (iv) refusal to participate.

The sample size estimation of this study follows the Kendall principle for sample size estimation, which states that the sample size should be at least 5 to 10 times the number of study variables (Kendall, 1957). After excluding the participant identification number and variables not used as independent variables, 18 candidate independent variables were included in this study, comprising 5 demographic and training characteristics, 4 hematological indicators, 5 physiological or training performance indicators, and 4 psychoperceptual indicators. Based on the lower bound of 10 participants per variable, a minimum of 180 participants was required. Considering an anticipated 10% rate of incomplete or invalid data, the minimum required sample size was 200 participants.

### 2.2 Measurements

#### 2.2.1 General information questionnaire

Demographic and training history data were collected after written assent and parental/guardian consent. A trained researcher completed a structured questionnaire and cross-checked entries against team registration records. Items included sex, age (years), training age (years of organized swimming), primary stroke, and sport level. Percentage of predicted adult height (%PAH) was calculated as current height divided by predicted adult height × 100 and was used as a noninvasive indicator of biological maturation status (Cumming et al., 2017).

#### 2.2.2 Dmax-derived lactate threshold intensity

Lactate threshold intensity was assessed using both a pool-based swimming incremental test and a land-based cycling incremental test. The pool-based test was used to obtain sport-specific threshold intensity, whereas the cycling test was used as a complementary land-based cardiorespiratory and metabolic assessment. In both tests, capillary blood lactate concentration was measured at rest and after each stage, and the relationship between blood lactate concentration and exercise intensity was used to determine the Dmax point. For both the swimming and cycling tests, the Dmax method was applied using the same calculation procedure. Briefly, the blood lactate concentration and exercise intensity data from all valid stages were fitted to generate an individual lactate intensity curve. A straight line was then drawn between the first and last points of the fitted curve. The Dmax point was defined as the point on the fitted curve with the greatest perpendicular distance from this straight line. The exercise intensity corresponding to this point was extracted as the threshold intensity. When the Dmax point fell between two observed stages, the corresponding intensity was estimated from the fitted curve. Blood lactate concentration at the Dmax point was also recorded as a descriptive marker of lactate response, but it was not used as the primary indicator for latent profile derivation.

The pool-based test consisted of a 5 × 200 m front crawl incremental protocol performed in a 50 m indoor pool (Beretić et al., 2025). Each 200 m repetition was completed within a 5 min block, including the swimming time and the post-exercise measurements. Swimming time was recorded for each 200 m repetition, and 50 m split times were also recorded to monitor pacing consistency. Immediately after each repetition, heart rate, rating of perceived exertion, mental fatigue score, and fingertip capillary blood lactate concentration were obtained in a standardized order. Swimming velocity for each repetition was calculated as distance divided by time. The Dmax point was identified from the blood lactate concentration and swimming velocity curve. The pool-based Dmax-derived lactate threshold intensity was expressed as the swimming velocity corresponding to the Dmax point relative to the swimmer’s event-specific personal best velocity, calculated as: pool-based lactate threshold intensity (%PB velocity) = swimming velocity at the Dmax point/personal best velocity × 100. Personal-best velocity was defined as the fastest 200-m front crawl performance (from official competition or testing) achieved during the 12 months prior to enrollment (May 2024 - April 2025), corresponding to the athlete’s best result for that competitive season.

The land-based cycling test was performed on a cycle ergometer (Monark 839 E; Monark Exercise AB, Vansbro, Sweden). The protocol started at 60 W and increased by 30 W every 3 min until volitional exhaustion. Breath by breath gas exchange was recorded continuously using a gas analysis system (MetaLyzer 3B; CORTEX Biophysik GmbH, Leipzig, Germany) (Fang and Bian, 2017). Capillary blood lactate concentration was measured at rest and at the end of each stage. The Dmax point was identified from the blood lactate concentration and cycling power output curve. The land-based Dmax-derived lactate threshold power was defined as the cycling power output corresponding to the Dmax point and was expressed in W. Absolute power was retained because the cycling protocol was prescribed and quantified in fixed watt increments, and the purpose of this indicator was to represent the external mechanical workload corresponding to the Dmax point under standardized land-based testing conditions. Thus, it was used as a modality-specific workload indicator rather than as a body-size-normalized measure of physiological capacity.

Lactate curves were considered physiologically plausible when they met the following criteria: (i) blood lactate concentration increased continuously with each successive exercise stage—curves showing a decrease or plateau (i.e., no net increase) between stages were excluded; (ii) the Dmax point fell between the second stage and the penultimate stage, excluding the first and last data points as potential Dmax locations (Cheng et al., 1992); (iii) at least five valid lactate measurements were available (excluding the resting baseline value) (Bentley et al., 2007). In addition to statistical standards, a visual inspection of the retained curves will be conducted to confirm there are no abnormal fluctuations or measurement errors. For the pool-based test, pacing was rigorously controlled using pool-side personnel who walked at the prescribed target velocity for each repetition; for the land-based test, the ergometer continuously monitored and displayed power output. Given this pacing control, blood lactate was expected to rise monotonically with each stage (Bentley et al., 2007); any decrease was therefore treated as a technical error (e.g., sampling or measurement error) and the corresponding test was excluded, because in conventional incremental protocols with progressively increasing workload there is no established physiological mechanism by which blood lactate concentration would decrease between successive stages (Bentley et al., 2007; Faude et al., 2009). Tests with incomplete stages, missing key blood lactate values, or curves that could not be fitted reliably were excluded from the analysis. The same algorithm and parameter settings were applied to the pool-based and land-based tests to ensure consistency across testing modalities. In swimming, Dmax-derived thresholds have demonstrated excellent test-retest reliability (ICC = 0.96–0.99) (Keskinen et al., 2010) and good agreement with maximal lactate steady state in competitive swimmers (Arsoniadis et al., 2024). In cycling, using an incremental protocol with 3-min stages, Dmax has shown good reproducibility (ICC = 0.903, SEM = 2.5 W) (Morton et al., 2012).

#### 2.2.3 Hematology (complete blood count)

Venous blood was drawn before testing at the Sports and Exercise Laboratory in the Science Building of the authors’ institution (Beijing, China). Samples were collected into EDTA-K2 tubes and analyzed the same day on an automated hematology analyzer (Mindray BC-6800; Shenzhen Mindray Bio-Medical Electronics Co., Ltd., Shenzhen, China). Daily internal quality control and manufacturer calibration procedures were followed.

#### 2.2.4 Psychoperceptual markers

Psychoperceptual markers included mean cycling rating of perceived exertion (RPE) across completed stages, mean swimming RPE across five 200 m repetitions, mean swimming mental fatigue across five 200 m repetitions, and sport mental toughness. Perceived exertion was assessed using the Borg CR10 scale (Borg, 1998), ranging from 0 (nothing at all) to 10 (maximal exertion). During the land-based cycling incremental test, RPE was recorded at the end of each completed 3-min stage. For each participant, the arithmetic mean of all stage-specific RPE scores obtained before test termination was calculated and operationalized as mean cycling RPE across completed stages. An incompletely performed stage was excluded from the calculation, and the number of completed stages contributing to the mean was recorded. During the pool-based 5 × 200 m front-crawl incremental test, RPE was recorded immediately after each 200 m repetition. The arithmetic mean of the five post-repetition RPE scores was calculated and operationalized as mean swimming RPE across five 200 m repetitions. Both variables were expressed as Borg CR10 scores, with higher values indicating greater average perceived exertion in the corresponding testing context.


$$\text{Mean cycling RPE}=\frac{\sum_{\text{j}=1}^{\text{m}}{\text{RPE}}_{\text{j}}}{\text{m}}$$

where m represents the number of completed cycling stages.


$$\text{Mean swimming RPE}=\frac{\sum_{\text{i}=1}^{5}{\text{RPE}}_{\text{i}}}{5}$$

Mental fatigue was assessed using a 100 mm visual analogue scale (VAS) (Coyne et al., 2021), anchored from 0 mm (no mental fatigue) to 100 mm (extreme mental fatigue). Mental fatigue was recorded before testing and immediately after each 200 m repetition during the pool-based incremental swimming test. For the present analyses, the arithmetic mean of the five post-repetition VAS scores was calculated and operationalized as mean swimming mental fatigue across five 200 m repetitions. Baseline measurements and mental-fatigue scores obtained during the cycling test were not included in this summary measure. The variable was expressed in millimetres, with higher values indicating greater average mental fatigue during the swimming test.


$$\text{Mean swimming mental fatigue}=\frac{\sum_{\text{i}=1}^{5}{\text{VAS}}_{\text{i}}}{5}$$

Sport mental toughness was measured at baseline using the Chinese version of the Sport Mental Toughness Questionnaire (Sheard et al., 2009). The questionnaire contains 12 items rated on a 5-point Likert scale, from 1 (strongly disagree) to 5 (strongly agree), with higher total scores indicating greater sport mental toughness. In the present sample, the internal consistency of the total scale was acceptable (Cronbach’s alpha = 0.74). The total score was used in the main analysis.

#### 2.2.5 Lactate-clearance half-time

Following the pool-based 5 × 200 m test, participants completed a 30-min passive seated recovery period. Fingertip capillary blood lactate concentration was measured immediately after the final repetition and at 3, 5, 7, 10, 15 min of recovery (Šiljeg et al., 2023). The highest observed post-exercise lactate concentration was defined as the peak value, and the peak and all subsequent measurements were fitted using a mono-exponential decay model:


$$La(t)=La_{\infty }+(La_{\text{peak}}-La_{\infty })e^{-k(t-t_{\text{peak}})}$$

where La(t) is the blood lactate concentration at time t, $La_{\text{peak}}$ is the observed peak concentration, $La_{\infty }$ is the estimated asymptotic concentration, and k is the decay-rate constant. Lactate-clearance half-time was calculated as $t_{1/2}=\text{ln}(2)/k$.

### 2.3 Procedure

All three centers followed the same testing schedule and pre test instructions. The land-based cycling test was performed first, followed by the pool-based swimming test 24 h later. The same fixed test order and 24-h interval were applied at all three centers to minimize potential acute carryover effects from the exhaustive cycling test.

**Figure 1:**
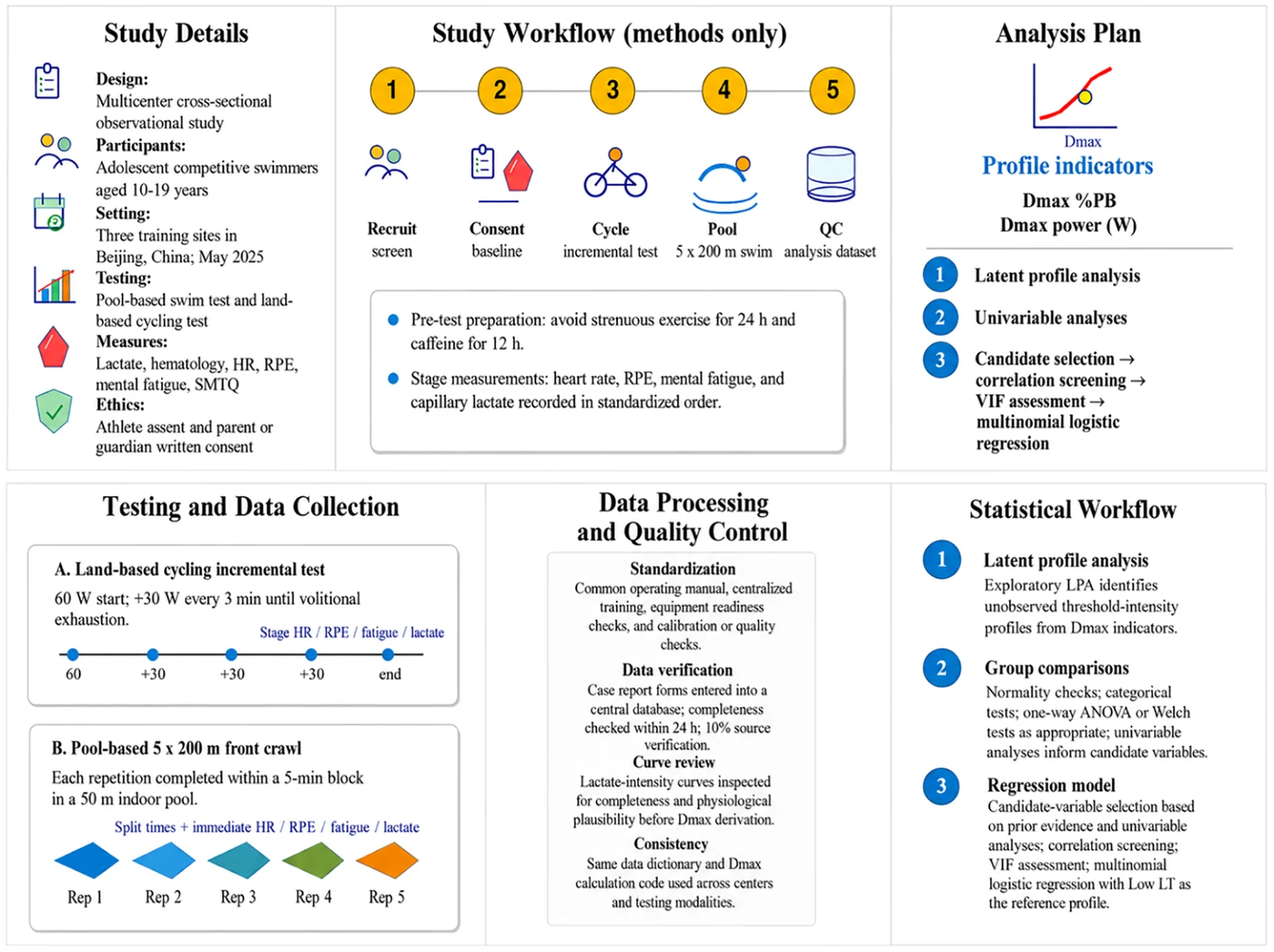
Overview of the study procedures.

Participants were asked to avoid strenuous exercise for 24 h and caffeine for 12 h before testing, and to arrive 2-3 h after a light meal in a well hydrated state. Upon arrival, eligibility was reconfirmed, and height and body mass were measured. Written assent from the swimmers and written informed consent from parents or guardians were obtained before any testing procedures. The overall study workflow is summarized in **Figure 1**.

After enrollment, participants completed the baseline questionnaire and the Sport Mental Toughness Questionnaire in a quiet room under standardized instructions. A resting fingertip capillary blood sample was then obtained for blood lactate measurement.

The land-based cycling incremental test was performed first, following a standardized warm-up. The test commenced at 60 W, with the workload increased by 30 W every 3 min until volitional exhaustion. Breath-by-breath gas-exchange and electrocardiographic data were recorded continuously throughout the test. At the end of each completed 3-min stage and before initiation of the subsequent workload, heart rate, rating of perceived exertion (RPE), mental fatigue, and capillary blood lactate concentration were assessed in a standardized order using identical instructions and assessment procedures across stages and study centers. RPE was assessed using the Borg CR10 scale, ranging from 0 to 10. For the present analyses, the RPE scores obtained at all completed stages were averaged for each participant and defined as mean cycling RPE across completed stages. An incompletely performed stage was not included in this calculation, and the number of completed stages contributing to the mean was recorded for quality-control purposes. After completion of the cycling test, participants followed the standardized recovery instructions, and the pool-based assessment was conducted 24 h later.

The pool-based 5×200 m front-crawl incremental test was performed after a standardized swimming warm-up. Participants completed five 200 m repetitions, each within a predefined 5 min block that included swimming and post-repetition measurements. Immediately after each repetition, measurements were obtained in a fixed sequence. Heart rate was counted during the first 10 s after completion of the repetition, followed by assessment of RPE using the Borg CR10 scale, mental fatigue using a 100 mm visual analogue scale, and fingertip capillary blood lactate concentration according to the standardized sampling protocol. After completion of all five repetitions, the five post-repetition RPE scores, mental-fatigue scores, and the five post-repetition 10 s heart-rate counts were averaged and defined as mean swimming RPE across five 200 m repetitions, mean swimming mental fatigue across five 200 m repetitions, and mean swimming heart rate across five 200 m repetitions, respectively. Baseline mental-fatigue measurements obtained before testing were not included in the five-repetition mean. Calculation of each summary measure required five valid post-repetition observations; participants with incomplete key measurements were handled according to the prespecified data-quality and exclusion criteria.

### 2.4 Data collection and quality control

Data were recorded using standardized case report forms with predefined variable names, units, value ranges, and mandatory fields. Each participant was assigned a unique study identification number, and all test results were recorded under this identifier. Field data, including swimming times, 50 m split times, heart rate, rating of perceived exertion, mental fatigue score, and blood lactate concentration, were recorded immediately after each relevant stage or repetition. Laboratory data, including hematological and lactate clearance variables, were merged into the central database using the same study identification number.

After data collection, paper forms were entered into a centralized electronic database by trained staff. Data completeness was checked within 24 h of testing, and 10% of records were independently verified against the original case report forms by a second researcher. Automated range and logic checks were used to identify implausible or inconsistent values, such as out of range rating of perceived exertion scores, inconsistent split time sums, missing lactate values, or physiologically implausible lactate responses. Any flagged values were resolved by reviewing the original records and, when necessary, by querying the corresponding testing center.

The same data dictionary and Dmax calculation code were used for all centers. Before statistical analysis, lactate intensity curves and derived Dmax variables were checked for completeness and physiological plausibility. Cases with incomplete testing procedures, missing key lactate or intensity data, or unreliable curve fitting were excluded according to predefined criteria. All data were deidentified before analysis and stored on a password protected server with access restricted to the research team.

### 2.5 Statistical analysis

Data were double-entered and checked. Normality was tested with Shapiro-Wilk (Shapiro and Wilk, 1965). Continuous data are reported as mean ± SD or median (IQR); categorical data as n (%). Latent profile models were estimated using robust maximum likelihood estimation (MLR). The two indicators were entered in their original measurement units. Indicator means were freely estimated across profiles, indicator variances were constrained to equality across profiles, and the within-profile covariance was fixed at zero. Models with one to five profiles were estimated using 400 initial-stage random starts and 100 final-stage optimizations. The retained three-profile model was rerun using 800 initial-stage random starts and 200 final-stage optimizations to verify solution stability. The default Mplus convergence criteria were used. A solution was considered acceptable when estimation terminated normally and the best log-likelihood value was replicated. A significant Lo-Mendell-Rubin test (P<0.05) supported the K-class model over the K−1 model, and higher entropy values (closer to 1) indicated better classification accuracy (Lo et al., 2001).

SPSS Statistics for Windows, Version 27.0 (IBM Corp., Armonk, NY, USA) was used for Pearson’s chi-square, Fisher’s exact, one-way ANOVA (Welch’s test when variances were unequal). Effect sizes were reported to complement statistical significance. For continuous variables, overall between-profile differences were expressed as ω² with 95% confidence intervals, whereas Cramér’s V with 95% confidence intervals was reported for categorical variables. To account for multiple testing across the univariable between-profile comparisons, the Benjamini-Hochberg procedure was applied to control the false discovery rate, and both unadjusted P values and FDR-adjusted q values were reported.

Candidate variables were selected *a priori* based on the literature and biological plausibility and supplemented by variables identified in the univariable analyses. Variables identified *a priori* were included in the candidate variable set irrespective of their univariable significance. Pairwise correlations among continuous candidate variables were examined, and when ∣r∣>.70, only the more conceptually relevant variable was retained (Dormann et al., 2013). The selected variables were subsequently entered into a multinomial logistic regression model, with the lower-threshold-intensity profile as the reference category. Study center was included in the multinomial logistic regression model as a categorical fixed effect to account for potential center-related differences. Two-sided P<0.05 was considered statistically significant (Zhang et al., 2023).

### 2.6 Ethical considerations

This study was approved by the Beijing Sport University Sports Science Experiment Ethics Committee of the authors’ institution (Approval No. 2024449H). The study purpose, procedures, potential risks, and benefits were explained before enrollment. Written informed consent was obtained from parents/guardians and written assent was obtained from adolescent swimmers. Participation was voluntary and could be withdrawn at any time without penalty. Data collection proceeded only with authorization, and all data were de-identified and stored securely. The study complied with the principles of the Declaration of Helsinki (World Medical Association, 2013).

## 3 Results

### 3.1 General characteristics of the participants

**Figure 2:**
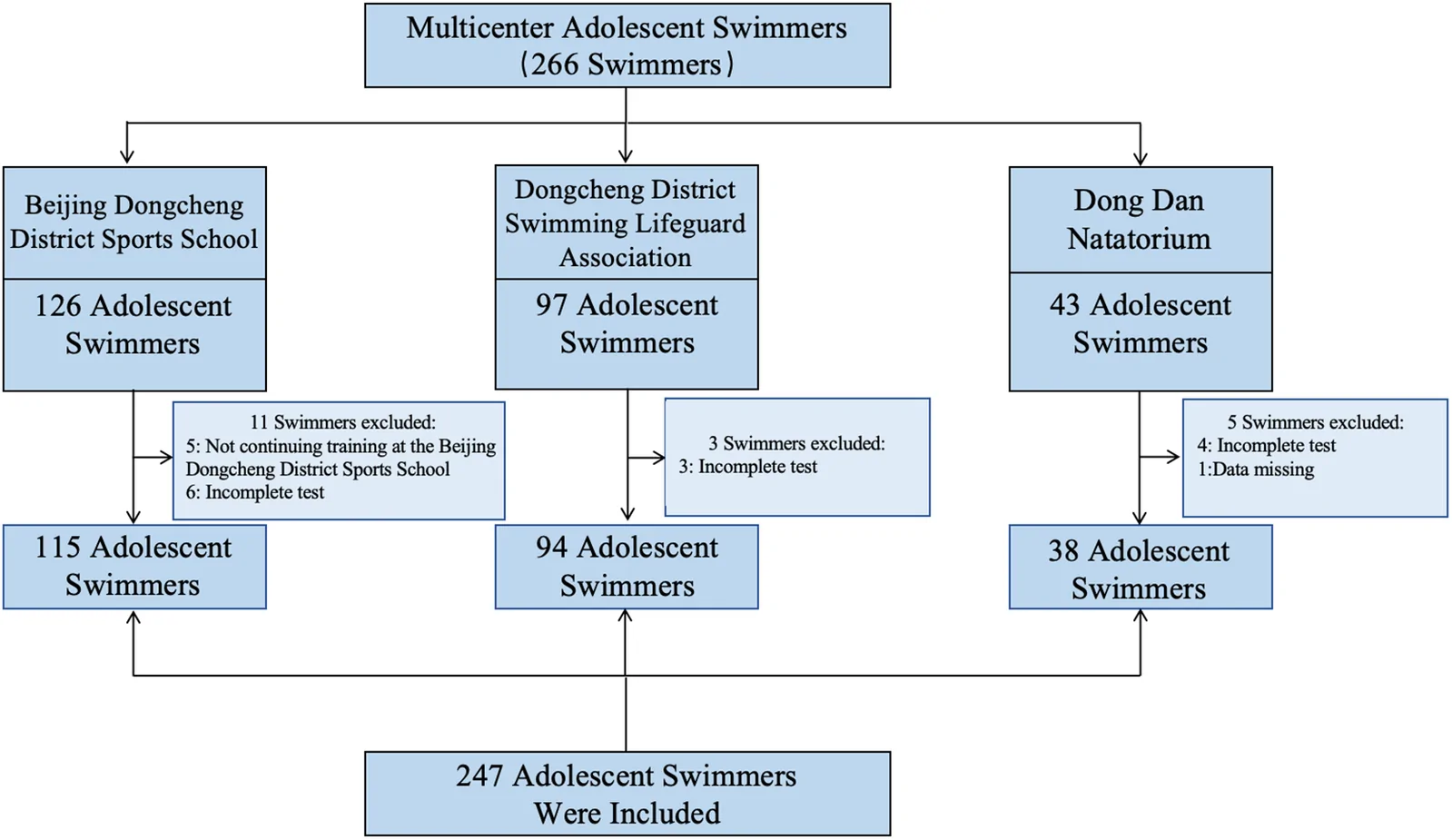
Flow chart of the study.

A total of 266 swimmers were screened in May 2025. 19 were excluded (five had discontinued team training, thirteen had incomplete incremental tests, and one had missing data), yielding a final sample of 247 adolescent swimmers (effective inclusion rate: 92.9%; **Figure 2**). In the retained sample, all lactate curves from both the pool-based and land-based incremental tests met the predefined physiological plausibility criteria, showing a continuous increase in blood lactate concentration across successive stages; therefore, no additional exclusions were made on the basis of non-monotonic lactate responses.

Participant characteristics and measured variables are presented in **Table 1**. The sample included 133 male swimmers (53.8%) and 114 female swimmers (46.2%). The mean age was 11.43 ± 2.61 years, the mean training age was 4.36 ± 2.36 years, and the mean body mass index was 18.69 ± 3.48 kg·m^-^². The overall Dmax-derived lactate-threshold intensity was 87.75 ± 1.82% of personal best velocity in the pool-based test, and the overall Dmax-derived lactate-threshold power was 124.87 ± 5.81 W in the land-based cycling test.

**Table 1:** Demographic and characteristics of study participants.

Variables	Category	Total(N = 247)
Gender	Male	133(53.8%)
	Female	114(46.2%)
Age (years)		11.43 ± 2.61
Sport Level	China first-class athlete	82(33.2%)
	China second-class athlete	142(57.5%)
	China third-class athlete	23(9.3%)
Training years (years)		4.36 ± 2.36
BMI (kg/m²)		18.69 ± 3.48
RBC (×10¹²/L)		4.92 ± 0.56
RDW (%)		13.48 ± 1.37
Platelets (×10^9^/L)		323.95 ± 58.90
PDW (%)		11.84 ± 2.43
Mean swimming heart rate across five 200 m repetitions (beats/10 s)		29.85 ± 1.85
Mean cycling RPE across completed stages (CR10, 0–10)		7.11 ± 1.61
%PAH (%)		82.69 ± 7.28
Last repetition completion (%PB)		95.18 ± 1.64
5x200 m average time (s)		170.42 ± 13.50
Mean swimming RPE across five 200 m repetitions (CR10, 0–10)		7.07 ± 0.64
Lactate clearance half-time (min)		17.15 ± 4.45
Mean swimming mental fatigue across five 200 m repetitions (VAS, mm)		38.86 ± 13.86
Sport mental toughness (SMTQ, score)		40.82 ± 6.41
Dmax lactate-threshold intensity (pool-based, %PB)		87.75 ± 1.82
Dmax lactate-threshold power (land-based cycling, W)		124.87 ± 5.81

BMI, Body Mass Index; RBC, Red Blood Cells; RDW, Red Cell Distribution Width; PDW, Platelet Distribution Width; RPE, Rating of Perceived Exertion; PAH, Percentage of Predicted Adult Height; VAS, Visual Analogue Scale; SMTQ, Sport Mental Toughness Questionnaire.

### 3.2 Latent profile analysis of Dmax-derived threshold intensity: model fit, class enumeration and labels

One to five profile latent profile models were fitted using the pool-based Dmax-derived lactate threshold intensity and the land-based Dmax-derived lactate threshold power as profile indicators. Both the bootstrap likelihood-ratio test and the Vuong-Lo-Mendell-Rubin test supported the three-profile model over the two-profile model. For models with four or more profiles, these tests did not indicate a statistically meaningful improvement over the three-profile solution. Considering model fit, parsimony, profile size, and interpretability, the three-profile model was therefore selected. For the retained three-profile model, the best log-likelihood value was successfully replicated and remained unchanged after the number of random starts was doubled, indicating that the retained solution was not attributable to a competing local maximum. The selected model also showed good classification accuracy, with an entropy value of 0.929 and average posterior probabilities of 0.998, 0.963, and 0.990 for the lower, intermediate-, and higher-threshold-intensity profiles, respectively. Results are shown in **Table 2**.

**Table 2:** Fit indices and classification quality for latent profile models of lactate-threshold intensity.

Class	LL	AIC	BIC	ABIC	Entropy	BLRT	VLMR	Class probability
						P-value	P-value	
1	-917.963	1843.926	1857.964	1845.284	–	–	–	–
2	-757.110	1528.220	1552.785	1530.595	0.885	0.002	0.002	43.32/56.68
3*	-678.067	1376.134	1411.227	1379.528	0.929	<0.001	<0.001	23.48/47.37/29.15
4	-656.745	1339.491	1385.113	1343.903	0.928	0.360	0.343	21.46/46.96/24.29/7.29
5	-637.440	1306.881	1363.031	1312.311	0.923	0.122	0.112	21.05/5.67/45.75/16.19/11.34

LL, Log Likelihood; AIC, Akaike Information Criterion; BIC, Bayesian Information Criterion; ABIC, Sample-size-adjusted Bayesian Information Criterion; BLRT, Bootstrapped Likelihood Ratio Test; VLMR, Vuong-Lo-Mendell-Rubin Likelihood Ratio Test; *: Best Fitting Model.

**Figure 3:**
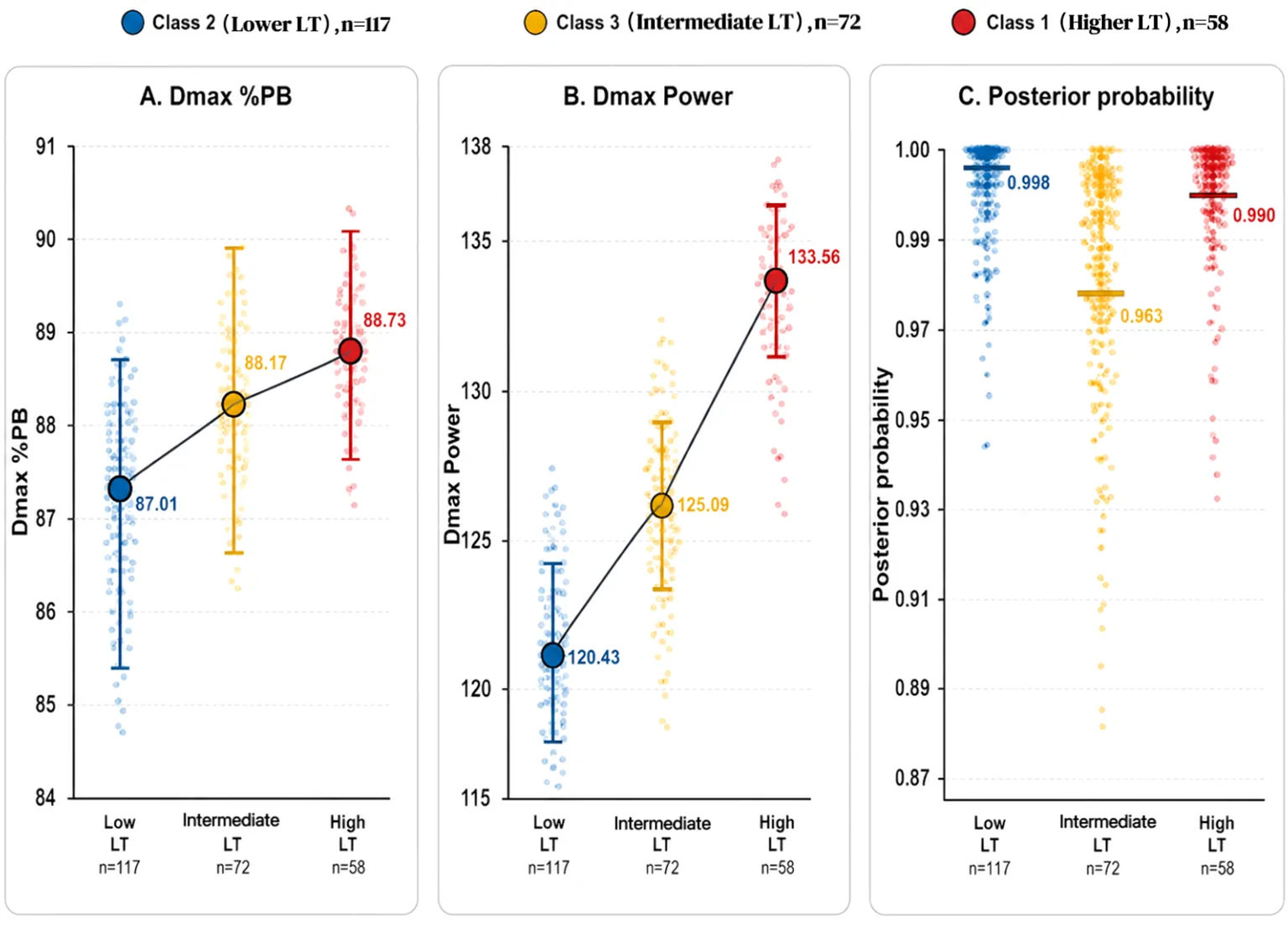
Individual Dmax-derived threshold-intensity indicators and posterior classification probabilities across the three latent profiles. Panels **(A, B)** show individual values for pool-based Dmax intensity relative to personal-best velocity and land-based cycling Dmax power, respectively. Small points represent individual participants, large points represent profile-specific means, and error bars represent 95% confidence intervals of the means. Panel **(C)** shows each participant’s posterior probability of membership in the assigned profile; horizontal lines indicate the profile-specific mean posterior probabilities. LT, lactate threshold; PB, personal-best velocity.

In the selected three-profile solution, Class 2 showed the lowest values for both Dmax-derived indicators and was labelled the lower lactate-threshold-intensity profile (n = 117, 47.4%; Dmax %PB velocity = 87.01%; Dmax power = 120.43 W). Class 3 showed intermediate values and was labelled the intermediate lactate-threshold-intensity profile (n = 72, 29.2%; Dmax %PB velocity = 88.17%; Dmax power = 125.09 W). Class 1 showed the highest values for both indicators and was labelled the higher lactate-threshold-intensity profile (n = 58, 23.5%; Dmax %PB velocity = 88.73%; Dmax power = 133.56 W). **Figure 3** presents the individual distributions of both Dmax-derived indicators, together with the profile-specific means and their 95% confidence intervals. Individual posterior probabilities of membership in the assigned profile were generally high, with mean probabilities of 0.998, 0.963, and 0.990 for the lower, intermediate, and higher profiles, respectively.

### 3.3 Univariate analysis across potential categories

After Benjamini-Hochberg false-discovery-rate correction, significant between-profile differences remained for red blood cell count, red cell distribution width, mean cycling RPE across completed stages, last-repetition completion relative to personal-best velocity, mean swimming RPE across five 200 m repetitions, lactate-clearance half-time, mean swimming mental fatigue across five 200 m repetitions, and sport mental toughness (all FDR-adjusted q <.05; **Table 3**). The largest between-profile effect was observed for mean swimming RPE across five 200 m repetitions (ω² = 0.646, 95% CI: 0.581–0.699), followed by mean cycling RPE across completed stages (ω² = 0.336, 95% CI: 0.247–0.421), red blood cell count (ω² = 0.295, 95% CI: 0.207–0.383), and last-repetition completion relative to personal-best velocity (ω² = 0.147, 95% CI: 0.077–0.233). The effects for red cell distribution width, lactate-clearance half-time, mean swimming mental fatigue across five 200 m repetitions, and sport mental toughness were comparatively small (ω² = 0.018–0.028). No significant between-profile differences were observed for sex, age, sport level, training age, body mass index, platelet count, platelet distribution width, mean swimming heart rate across five 200 m repetitions, percentage of predicted adult height, or mean 5 × 200 m completion time after FDR correction (all q > 0.05). Profile distribution also did not differ across the three study centers (q = 0.998; Cramér’s V = 0.019, 95% CI: <0.001–0.047).

**Table 3:** Comparisons of participant characteristics and measured variables across lactate-threshold intensity profiles (N = 247).

Variable	Lower lactate threshold group (N = 117)	Intermediate lactate threshold group (N = 72)	Higher lactate threshold group (N = 58)	Test statistic	P value	FDR-adjusted q	Effect size (95% CI)
Gender				χ² = 2.377	0.305	0.475	V = 0.098 (<0.001, 0.208)
Male	69 (59.0%)	35 (48.6%)	29 (50.0%)				
Female	48 (41.0%)	37 (51.4%)	29 (50.0%)				
Age(year)	11.18 ± 2.49	11.71 ± 2.87	11.59 ± 2.49	F = 1.055	0.350	0.475	ω² = 0.0004 (<0.001, 0.039)
Sport Level				χ² = 2.252	0.690	0.819	V = 0.068 (<0.001, 0.121)
China first-class athlete	40 (34.2%)	25 (34.7%)	17 (29.3%)				
China second-class athlete	67 (57.3%)	38 (52.8%)	37 (63.8%)				
China third-class athlete	10 (8.5%)	9 (12.5%)	4 (6.9%)				
Training Years(year)	4.13 ± 2.24	4.54 ± 2.36	4.60 ± 2.60	F = 1.083	0.340	0.458	ω² = 0.0007 (<0.001, 0.040)
BMI(kg/m²)	18.34 ± 3.10	18.89 ± 3.56	19.16 ± 4.06	F = 1.250	0.288	0.437	ω² = 0.0020 (<0.001, 0.043)
**RBC(×10¹²/L)**	**4.63 ± 0.53**	**5.07 ± 0.47**	**5.33 ± 0.37**	**F = 52.677**	**<0.001**	**<0.001**	**ω² = 0.295 (0.207, 0.383)**
**RDW (%)**	**13.38 ± 1.44**	**13.33 ± 1.28**	**13.88 ± 1.28**	**F = 3.290**	**0.039**	**0.047**	**ω² = 0.018 (<0.001, 0.072)**
Platelets(×10^9^/L)	321.50 ± 56.15	326.68 ± 58.68	325.48 ± 65.15	F = 0.197	0.822	0.919	ω² = <0.001 (<0.001, 0.017)
PDW	11.82 ± 2.15	11.82 ± 3.07	11.88 ± 2.09	F = 0.015	0.985	0.997	ω² = <0.001 (<0.001, <0.001)
Mean swimming heart rate across five 200m repetitions (beats/10 s)	30.04 ± 1.80	29.49 ± 1.87	29.93 ± 1.90	F = 2.108	0.124	0.262	ω² = 0.0089 (<0.001, 0.056)
**Mean cycling RPE across completed stages (CR10, 0–10**)	**7.99 ± 1.45**	**6.68 ± 1.44**	**5.88 ± 1.00**	**F = 63.440**	**<0.001**	**<0.001**	**ω² = 0.336 (0.247, 0.421)**
%PAH(%)	82.03 ± 6.60	83.62 ± 7.91	82.85 ± 7.75	F = 1.083	0.340	0.475	ω² = 0.0007 (<0.001, 0.040)
**Last repetition completion (%PB)**	**94.57 ± 1.62**	**95.36 ± 1.50**	**96.18 ± 1.32**	**F = 22.281**	**<0.001**	**<0.001**	**ω² = 0.147 (0.077, 0.233)**
5x200 m average time (s)	171.20 ± 12.55	169.08 ± 15.72	170.50 ± 12.42	F = 0.549	0.578	0.732	ω² = <0.001 (<0.001, 0.029)
**Mean swimming RPE across five 200m repetitions (CR10, 0–10)**	**7.57 ± 0.38**	**6.88 ± 0.36**	**6.32 ± 0.40**	**F = 226.118**	**<0.001**	**<0.001**	**ω² = 0.646 (0.581, 0.699)**
**Lactate clearance half-time (min)**	**17.85 ± 4.63**	**16.91 ± 4.25**	**16.04 ± 4.11**	**F = 3.428**	**0.034**	**0.049**	**ω² = 0.019 (<0.001, 0.074)**
**Mean swimming mental fatigue across five 200m repetitions (VAS, mm)**	**41.22 ± 14.72**	**37.98 ± 12.79**	**35.22 ± 12.58**	**F = 3.933**	**0.021**	**0.035**	**ω² = 0.023 (0.0004, 0.080)**
**Sport mental toughness (SMTQ, score)**	**39.70 ± 6.83**	**41.11 ± 6.12**	**42.73 ± 5.38**	**F = 4.588**	**0.011**	**0.042**	**ω² = 0.028 (0.0020, 0.088)**
Center				χ² = 0.171	0.997	0.998	V = 0.019 (<0.001, 0.047)
1	40 (34.2%)	26 (36.1%)	20 (34.5%)				
2	45 (38.5%)	28 (38.9%)	22 (37.9%)				
3	32 (27.4%)	18 (25.0%)	16 (27.6%)				

Data are presented as mean ± SD for continuous variables and *n* (%) for categorical variables. *P* values from one-way ANOVA (Welch's test when Levene's test *P* < 0.05) for continuous variables and Pearson's chi-square test for categorical variables. FDR-adjusted *q* values from the Benjamini–Hochberg procedure across all *m* = 19 comparisons. *ω²* = omega squared; *V* = Cramér's *V*. 95% confidence intervals from the non-central *F* (continuous) or non-central *χ²* distribution.*P < 0.05; **P < 0.01; ***P < 0.001 (unadjusted). BMI, body mass index; RBC, red blood cell count; RDW, red cell distribution width; PDW, platelet distribution width; HR, heart rate; RPE, rating of perceived exertion; %PAH, percentage of predicted adult height; %PB, percentage of personal best velocity; SMTQ, Sport Mental Toughness Questionnaire; VAS, visual analogue scale; LT, lactate threshold. Center 1: Beijing Dongcheng District Sports School, Center 2: Dongcheng District Swimming Lifeguard Association, Center 3: Dong Dan natatorium. Rows highlighted in bold indicate variables with p < 0.05.

Red blood cell count and last-repetition completion relative to personal-best velocity increased from the lower- to the higher-threshold-intensity profile. In contrast, both mean cycling RPE across completed stages and mean swimming RPE across five 200 m repetitions decreased progressively across the profiles, with the lowest values observed in the higher-threshold-intensity profile. Mean swimming mental fatigue across five 200 m repetitions and lactate-clearance half-time also decreased across the ordered profiles, whereas sport mental toughness increased. Red cell distribution width was highest in the higher-threshold-intensity profile, although the magnitude of the between-profile difference was small.

### 3.4 Selection of variables for multivariable analysis of latent profiles

**Figure 4:**
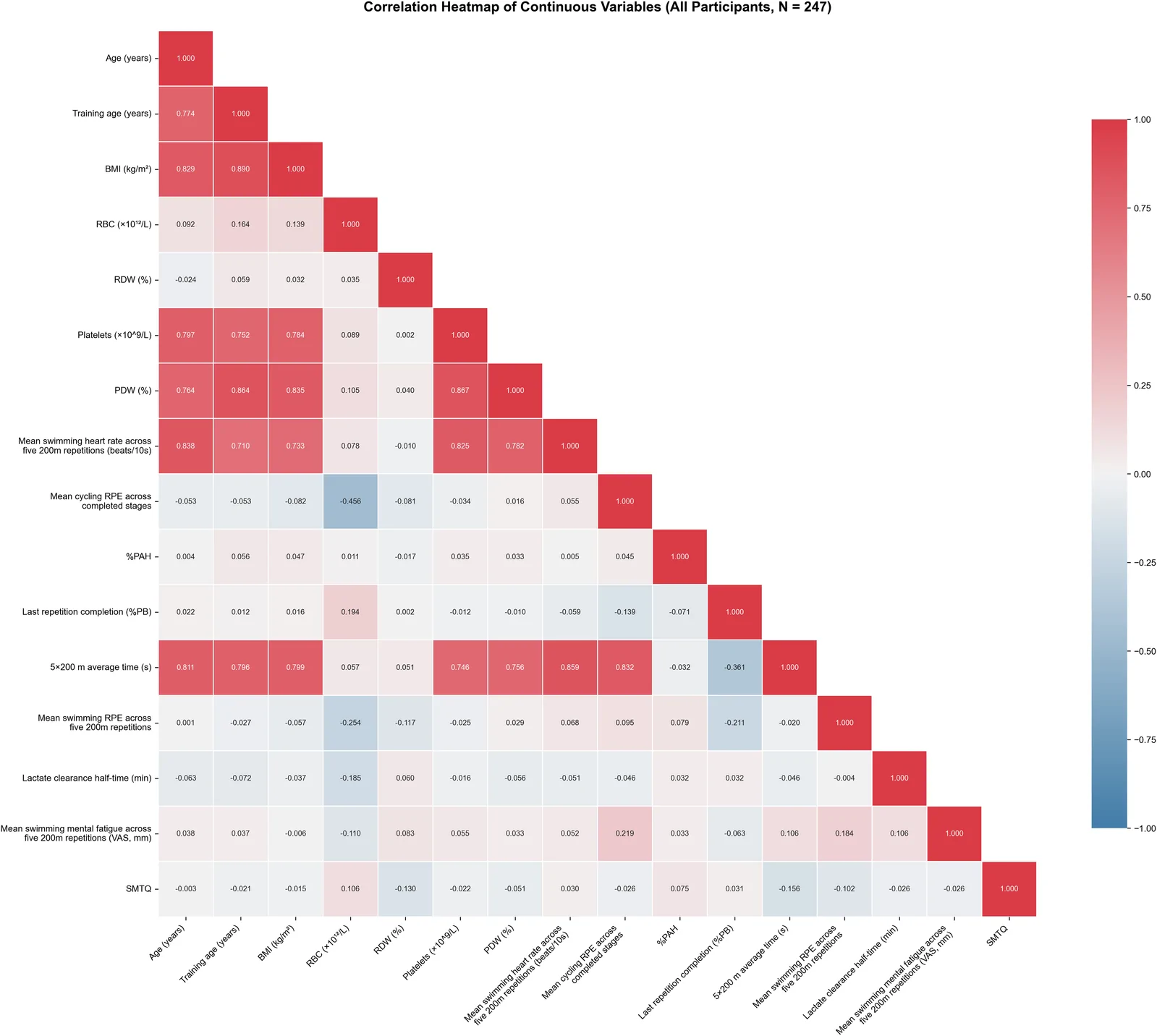
Correlation heatmap of continuous candidate variables.

Pairwise Pearson correlations among the continuous candidate variables are presented in **Figure 4**. Twenty-two variable pairs showed strong correlations (∣r∣>0.70). Strong positive correlations were observed among age, training age, body mass index, platelet count, platelet distribution width, mean swimming heart rate across five 200 m repetitions, mean 5 × 200 m completion time, with correlation coefficients ranging from r=0.710 to r=0.890. The strongest correlation was observed between training age and body mass index (r=0.890). 5×200 m average time was strongly correlated with mean cycling RPE across completed stages (r=0.832).

Within sets of highly correlated candidate variables, the variable with greater conceptual relevance and interpretability was retained. Age, training age, body mass index, platelet count, platelet distribution width, mean swimming heart rate across five 200 m repetitions, mean 5 × 200 m completion time, and mean cycling RPE across completed stages were excluded during correlation screening. Red blood cell count, red cell distribution width, mean swimming RPE across five 200 m repetitions, last-repetition completion relative to personal-best velocity, percentage of predicted adult height, lactate-clearance half-time, mean swimming mental fatigue across five 200 m repetitions, and sport mental toughness were retained. The VIF values of the retained continuous predictors ranged from 1.020 to 1.166, with the highest value observed for mean swimming RPE across five 200 m repetitions and the lowest for percentage of predicted adult height. All VIF values were below 2, indicating no substantial multicollinearity among the predictors included in the final model (**Table 4**).

**Table 4:** Variance inflation factors for predictors in the multinomial logistic regression model.

Variable	VIF
RBC (×10¹²/L)	1.150
RDW (%)	1.055
Mean swimming RPE across five 200 m repetitions (CR10, 0–10)	1.166
Last repetition completion (%PB)	1.089
%PAH (%)	1.020
Lactate clearance half-time (min)	1.051
Mean swimming mental fatigue across five 200-m repetitions (VAS mm)	1.077
Sport mental toughness (SMTQ, score)	1.059

VIF < 5: low multicollinearity (Hair et al., 2019). All predictors exhibit low multicollinearity (VIF < 2).

### 3.5 Multivariate analysis of factors associated with potential categories

Following candidate-variable selection, correlation screening, and multicollinearity assessment, red blood cell count, red cell distribution width, mean swimming RPE across five 200 m repetitions, last-repetition completion relative to personal-best velocity, percentage of predicted adult height, lactate-clearance half-time, mean swimming mental fatigue across five 200 m repetitions, and sport mental toughness were entered into the multinomial logistic regression model. Study center was included as prespecified adjustment covariate for potential center-related differences. Lactate-threshold-intensity profile membership was specified as the dependent variable, with the lower-threshold-intensity profile as the reference category. The results are presented in **Table 5**.

**Table 5:** Multinomial logistic regression of factors associated with lactate-threshold intensity profile.

Variables	Intermediate LT group				Higher LT group			
	β	OR	95%CI	P	β	OR	95%CI	P
**RBC (×10¹²/L)**	**2.050**	**7.765**	**(3.198**, **18.849)**	**<0.001**	**3.699**	**40.412**	**(10.844**, **150.605)**	**<0.001**
RDW (%)	0.002	1.002	(0.746, 1.345)	0.990	0.255	1.290	(0.860, 1.936)	0.218
**Mean swimming RPE across five 200 m repetitions (CR10**, **0–10)**	**-0.693**	**0.500**	**(0.372**, **0.672)**	**<0.001**	**-1.203**	**0.300**	**(0.197**, **0.457)**	**<0.001**
**Last repetition completion (%PB)**	**0.545**	**1.725**	**(1.317**, **2.259)**	**<0.001**	**1.066**	**2.903**	**(2.007**, **4.200)**	**<0.001**
%PAH (%)	0.006	1.006	(0.952, 1.063)	0.830	-0.063	0.939	(0.872, 1.011)	0.093
Lactate clearance half-time (min)	-0.091	0.913	(0.827, 1.007)	0.069	-0.076	0.926	(0.808, 1.062)	0.274
**Mean swimming mental fatigue across five 200 m repetitions (VAS mm)**	-0.029	0.971	(0.943, 1.001)	0.059	**-0.047**	**0.954**	**(0.916**, **0.993)**	**0.022**
**Sport mental toughness (SMTQ**, **score)**	0.038	1.038	(0.979, 1.101)	0.210	**0.125**	**1.134**	**(1.041**, **1.235)**	**0.004**

LT, Lactate Threshold. Rows highlighted in bold indicate coefficients with p < 0.05 in at least one comparison. Reference category: Lower Lactate Threshold group. Study center was included as prespecified adjustment covariate.

Compared with the lower-threshold-intensity profile, higher red blood cell count was associated with greater odds of membership in both the intermediate profile (OR = 7.765, 95% CI: 3.198–18.849, P <.001) and the higher profile (OR = 40.412, 95% CI: 10.844–150.605, P <.001). Higher mean swimming RPE across five 200 m repetitions was associated with lower odds of membership in the intermediate profile (OR = 0.500, 95% CI: 0.372–0.672, P <.001) and the higher profile (OR = 0.300, 95% CI: 0.197–0.457, P <.001). In contrast, higher last-repetition completion relative to personal-best velocity was associated with greater odds of membership in both the intermediate profile (OR = 1.725, 95% CI: 1.317–2.259, P <.001) and the higher profile (OR = 2.903, 95% CI: 2.007–4.200, P <.001). Lower mean swimming mental fatigue across five 200 m repetitions was associated with membership in the higher profile only (OR = 0.954, 95% CI: 0.916–0.993, P = .022), whereas higher sport mental toughness was positively associated with membership in the higher profile only (OR = 1.134, 95% CI: 1.041–1.235, P = .004). Red cell distribution width, percentage of predicted adult height, and lactate-clearance half-time were not independently associated with membership in either the intermediate or higher profile.

## 4 Discussion

### 4.1 Threshold intensity stratification and training interpretation

This study identified three data-driven, ordered lactate-threshold-intensity profiles in adolescent swimmers using pool-based Dmax percentage of personal-best velocity and land-based Dmax cycling power. The lower, intermediate, and higher profiles showed an ordered increase across both indicators, reflecting relative differences in the intensity at which threshold-related changes occurred within the present sample. This finding supports the value of describing youth swimmers through threshold-intensity patterns rather than through group averages alone, because athletes with similar age, training background, or mean test performance may still differ in their ability to sustain intensity near the threshold domain (Armstrong and Welsman, 1994; Keller et al., 2023).

The lower-threshold-intensity profile may represent swimmers whose threshold-related transition occurs at a relatively lower percentage of personal best velocity and lower cycling power. In training practice, this pattern may prompt further assessment of aerobic capacity, pacing control, and technical efficiency (Toubekis and Tokmakidis, 2013).

The intermediate-threshold-intensity profile represented an intermediate position in the joint distribution of the two Dmax-derived indicators. From a practical perspective, this pattern may prompt closer attention to threshold-pace control, responses across progressively demanding intervals, and the maintenance of swimming technique under fatigue. Longitudinal studies with repeated testing are needed to determine whether profile membership remains stable or changes in response to training, and whether within-athlete changes in threshold-intensity profiles are accompanied by corresponding changes in aerobic capacity, lactate handling, and swimming-specific efficiency.

The higher threshold intensity profile was characterized by a higher relative swimming velocity at the Dmax point and higher land-based threshold power. This profile may indicate stronger tolerance to threshold intensity and better integration between physiological capacity and task execution. In practical terms, swimmers showing this pattern may be able to sustain threshold-related exercise at a higher relative intensity with lower perceived strain. This profile may therefore prompt further evaluation of how effectively this threshold-related capacity is expressed through event-specific pace control, race rhythm, and the ability to reproduce high-quality efforts under repeated-exercise conditions (Nikitakis et al., 2025).

### 4.2 Oxygen transport, relative intensity tolerance, and metabolic recovery across lactate threshold intensity profiles

Red blood cell count was higher across the ordered profiles and was positively associated with membership in the intermediate- and higher-threshold-intensity profiles. This association may indicate a relationship between hematological oxygen-transport characteristics and Dmax-derived threshold intensity. A greater red blood cell count may support arterial oxygen delivery during progressive exercise, allowing a larger proportion of energy demand to be met through oxidative metabolism before the rapid rise in lactate concentration (Montero et al., 2015). From a training perspective, basic hematological monitoring may provide complementary context when interpreting threshold-intensity tests, particularly during periods of high training load, rapid growth, or intensified competition preparation, although its practical contribution requires further validation.

The last 200 m repetition completed relative to personal best velocity increased from the lower to the higher profile and was strongly associated with profile membership. This variable has clear practical meaning because it reflects the swimmer’s ability to maintain a high relative intensity at the end of a repeated 200 m set. A higher final-repetition percentage may be consistent with greater fatigue resistance or pacing stability, although these mechanisms were not directly assessed. In daily training, final-repetition velocity relative to personal-best velocity may be interpreted alongside lactate responses and perceived exertion to provide a more comprehensive assessment of repeated-set performance (Espada et al., 2021). Lactate-clearance half-time was shorter in the intermediate- and higher-threshold-intensity profiles in the univariable comparisons but was not associated after adjustment for the other model variables and study center. This attenuation suggests that the observed between-profile differences in lactate-clearance half-time may partly reflect shared variance with other physiological, performance-related, and perceptual characteristics rather than a unique association with profile membership. A shorter lactate-clearance half-time may nevertheless be consistent with more effective lactate transport, oxidation, and redistribution during recovery (Nikitakis and Toubekis, 2026), which may be relevant to swimmers performing repeated high-quality efforts with short recovery intervals. However, given the absence of an independent association, lactate-clearance half-time should currently be regarded as a complementary contextual marker rather than a direct basis for prescribing set structure, recovery duration, or active recovery intensity. Future studies are needed to determine whether integrating lactate-clearance measures with performance and perceptual indicators improves the monitoring of repeated-effort capacity and training adaptation.

### 4.3 Perceptual regulation and psychological readiness around threshold intensity

The higher-threshold-intensity profile combined higher threshold-intensity indicators with lower reported perceived exertion. Swimmers in this profile reached a greater relative swimming intensity and higher land-based threshold power while reporting lower mean swimming RPE across the five 200 m repetitions. This pattern indicates that the profiles differed not only in threshold-intensity indicators but also in concurrently measured perceived exertion. In swimming, perceived exertion is shaped by multiple factors, including breathing rhythm, pacing control, stroke efficiency, discomfort tolerance, and accumulated fatigue (Wallace et al., 2009). Sustaining a higher relative velocity with lower perceived exertion may reflect more favorable integration of physiological capacity, technical execution, and task-related responses near the threshold domain.

This finding may have potential relevance for training monitoring. Relative velocity or power alone cannot fully explain how demanding a set is for an individual swimmer. Two athletes may complete the same relative intensity, but the athlete reporting a higher perceived exertion may be experiencing greater internal strain, less efficient pacing, or poorer technical stability. Combining threshold-intensity indicators with perceived exertion may provide a more complete description of external and internal responses than either measure alone. In practice, RPE may provide useful complementary information when repeated threshold or cruise-pace sets are interpreted alongside split times and lactate responses (Seifert and Carmigniani, 2023).

Mental fatigue adds another layer to the interpretation of threshold-intensity profiles. Lower mental fatigue was associated with membership in the higher-threshold-intensity profile after adjustment for the other model variables and study center, which may reflect differences in concurrent psychological readiness during demanding repeated efforts. For adolescent swimmers, mental fatigue is closely connected with attention control, pacing judgment, response to discomfort, and the ability to maintain technique under pressure. Previous research has demonstrated that mental fatigue impairs both cognitive and aerobic performance in adolescent endurance athletes (Slimani et al., 2018). Academic workload, insufficient sleep, dense training schedules, and competition stress may all elevate mental fatigue and make the same physiological intensity feel more difficult (Habay et al., 2023). Mental fatigue may therefore provide useful contextual information when threshold intensity and perceived exertion are interpreted together.

Higher sport mental toughness was associated with membership in the higher-threshold-intensity profile after adjustment for the other model variables and study center and may be related to greater confidence, persistence, emotional control, and sustained task focus during demanding threshold-related efforts (Hsieh et al., 2024). These characteristics may influence how athletes regulate effort and maintain task execution under fatigue, providing a potential psychological dimension to the interpretation of threshold-intensity profiles (Reinebo et al., 2024). Future intervention studies should examine whether psychological-skills strategies, such as goal setting, cue-based self-talk, breathing-rhythm regulation, and feedback on pacing consistency, influence technical maintenance, effort regulation, perceived exertion, mental fatigue, or threshold-related performance.

Taken together, the psychological and perceptual findings suggest that lactate threshold intensity profiles may be interpreted not only through physiological capacity, but also through the athlete’s internal experience of effort and readiness. For adolescent swimmers, whose training response is influenced by rapid growth, school demands, and developing self regulation ability (Mihailescu et al., 2021), brief monitoring of perceived exertion, mental fatigue, and psychological readiness may strengthen the practical interpretation of threshold testing. These markers may provide coaches with complementary contextual information when interpreting an athlete’s threshold-test performance, including whether the observed response may reflect adequate adaptation, transient fatigue, or a mismatch between external load and internal tolerance.

### 4.4 Practical applications

The identified profiles may provide coaches with an exploratory framework for interpreting threshold-intensity testing at the individual level. Pool-based Dmax intensity provides the primary sport-specific reference, whereas cycling-derived Dmax power offers complementary information about threshold-related capacity under a standardized land-based workload. Considered together, these two indicators may provide a broader view of individual threshold-intensity characteristics than either measure alone.

In practice, threshold-test results may be interpreted alongside final-repetition velocity relative to personal-best velocity, perceived exertion, mental fatigue, and relevant hematological information. Considering external performance together with internal responses may help distinguish a relatively high threshold intensity accompanied by manageable perceived strain from a similar external performance accompanied by greater internal strain or transient fatigue. Brief assessment of perceived exertion and mental fatigue may therefore provide useful contextual information when interpreting test performance, particularly during periods of rapid growth, high academic demand, intensified training, or competition preparation. Repeated assessments conducted under comparable conditions may further help coaches interpret within-athlete changes in relation to training history, maturation, technical performance, and current readiness. In this context, profile membership may serve as a supplementary framework for individualized monitoring and interpretation rather than as a stand-alone basis for prescribing specific training interventions.

## 5 Conclusions

This study identified three data-driven, ordered Dmax-derived lactate-threshold-intensity profiles among adolescent swimmers by integrating pool-based threshold intensity relative to personal-best velocity and land-based cycling threshold power. The lower, intermediate, and higher profiles showed ordered differences in both threshold-intensity indicators, indicating relative heterogeneity within the present sample. However, the practical significance of the absolute between-profile differences requires further evaluation. The higher lactate-threshold intensity profile was characterized by a higher red blood cell count, greater final repetition completion relative to personal-best velocity, lower perceived exertion, lower mental fatigue, and greater sport mental toughness. Overall, integrating Dmax-derived threshold-intensity indicators with hematological, performance-related, and psychoperceptual markers may enhance the interpretation of individual threshold-test responses and support more individualized monitoring and assessment in training practice. However, these profiles should remain an exploratory assessment framework rather than prescriptive training categories until their reliability, longitudinal stability, and responsiveness to training have been established.

## 6 Limitations

This study has several limitations. As a multicenter cross-sectional observational study, several performance-related and psychoperceptual variables were measured during the same testing procedures used to derive the profile indicators. These associations should therefore be interpreted as concurrent test-related characteristics rather than temporally independent predictors. Although this study identified associations between lactate-threshold intensity profiles and physiological, performance-related, and psychoperceptual markers, it cannot establish causal relationships. Participants were recruited by convenience sampling from three training centers in Beijing, which may limit the generalizability of the findings to adolescent swimmers from other regions, training systems, or competitive levels. Although both pool-based and land-based tests were used, the cycling test is not swimming-specific and should be interpreted as a complementary assessment of threshold-related exercise capacity rather than a direct substitute for pool-based threshold testing. In addition, cycling-derived Dmax power was expressed in absolute watts and may therefore partly reflect differences in body size and maturation within this adolescent cohort; accordingly, it should be interpreted as the absolute external workload achieved at the Dmax point rather than as a body-size-independent index of physiological capacity. Future studies should include larger and more diverse samples, longitudinal follow-up, repeated testing, and more comprehensive maturation measures to examine the stability, predictive validity, and practical applicability of Dmax-derived lactate-threshold intensity profiles in youth swimming training.

Finally, the latent profiles have not been externally validated in an independent sample, and the test-retest reliability and measurement error of the Dmax-derived variables were not assessed. Given the relatively modest absolute separation between profiles, particularly in swimming-derived Dmax percentage of personal-best velocity between the intermediate and higher profiles, and the fact that the profiles were derived from only two monotonically ordered continuous indicators, the present findings cannot establish naturally discrete or qualitatively distinct physiological phenotypes. Replication using repeated measurements, additional indicators, and independent samples is warranted.

## Data Availability

The datasets presented in this article are not readily available because they involve detailed kinematic and physiological information of key athletes and cannot be fully disclosed. If there is a related need, partial anonymized original data can be provided. Requests to access the datasets should be directed to JLy, jiayi2025@bsu.edu.cn.
